# Unlocking Precision Medicine: Liquid Biopsy Advancements in Renal Cancer Detection and Monitoring

**DOI:** 10.3390/ijms25073867

**Published:** 2024-03-30

**Authors:** Felice Crocetto, Alfonso Falcone, Benito Fabio Mirto, Enrico Sicignano, Giovanni Pagano, Fabrizio Dinacci, Domenico Varriale, Fabio Machiella, Gaetano Giampaglia, Armando Calogero, Filippo Varlese, Raffaele Balsamo, Francesco Trama, Antonella Sciarra, Francesco Del Giudice, Gian Maria Busetto, Matteo Ferro, Giuseppe Lucarelli, Francesco Lasorsa, Ciro Imbimbo, Biagio Barone

**Affiliations:** 1 Department of Neuroscience, Reproductive Sciences and Dentistry, University of Naples “Federico II”, 80131 Naples, Italy; felice.crocetto@unina.it (F.C.); alfonso.falcone01@gmail.com (A.F.); fmirto22@gmail.com (B.F.M.); enrisici90@gmail.com (E.S.); giovanni.pagano1@outlook.it (G.P.); fabriziodinacci18@gmail.com (F.D.); domenicov93@libero.it (D.V.); f.machiella@gmail.com (F.M.); gaetanogiampaglia@hotmail.it (G.G.); ciro.imbimbo@unina.it (C.I.); 2 Department of Advanced Biomedical Science, University of Naples “Federico II”, 80131 Naples, Italy; calogero2@unina.it (A.C.); filippovarlese85@gmail.com (F.V.); 3 Urology Unit, Monaldi Hospital, 80131 Naples, Italy; raffaelebalsamo5@gmail.com; 4 ASL Napoli 2 Nord, P.O. Santa Maria delle Grazie, 80078 Pozzuoli, Italy; francescotrama@gmail.com; 5 Department of Experimental Medicine, University of Campania “Luigi Vanvitelli”, 80138 Naples, Italy; antonella.sciarra@unicampania.it; 6 Department of Maternal Infant and Urological Sciences, Umberto I Polyclinic Hospital, Sapienza University, 00161 Rome, Italy; francesco.delgiudice@uniroma1.it; 7 Department of Urology and Renal Transplantation, University of Foggia, 71122 Foggia, Italy; gianmaria.busetto@unifg.it; 8 Division of Urology, European Institute of Oncology (IEO)-IRCCS, 20141 Milan, Italy; matteo.ferro@ieo.it; 9 Urology, Andrology and Kidney Transplantation Unit, Department of Precision and Regenerative Medicine and Ionian Area, University of Bari “Aldo Moro”, 70124 Bari, Italy; giuseppe.lucarelli@inwind.it (G.L.); francesco-lasorsa96@libero.it (F.L.); 10 Urology Unit, Department of Surgical Sciences, AORN Sant’Anna e San Sebastiano, 81100 Caserta, Italy

**Keywords:** renal cell carcinoma, liquid biopsy, circulating tumor cells, microRNA, exosomes, diagnosis, prognosis

## Abstract

Renal cell carcinoma (RCC) remains a formidable diagnostic challenge, especially in the context of small renal masses. The quest for non-invasive screening tools and biomarkers has steered research towards liquid biopsy, focusing on microRNAs (miRNAs), exosomes, and circulating tumor cells (CTCs). MiRNAs, small non-coding RNAs, exhibit notable dysregulation in RCC, offering promising avenues for diagnosis and prognosis. Studies underscore their potential across various biofluids, including plasma, serum, and urine, for RCC detection and subtype characterization. Encouraging miRNA signatures show correlations with overall survival, indicative of their future relevance in RCC management. Exosomes, with their diverse molecular cargo, including miRNAs, emerge as enticing biomarkers, while CTCs, emanating from primary tumors into the bloodstream, provide valuable insights into cancer progression. Despite these advancements, clinical translation necessitates further validation and standardization, encompassing larger-scale studies and robust evidence generation. Currently lacking approved diagnostic assays for renal cancer, the potential future applications of liquid biopsy in follow-up care, treatment selection, and outcome prediction in RCC patients are profound. This review aims to discuss and highlight recent advancements in liquid biopsy for RCC, exploring their strengths and weaknesses in the comprehensive management of this disease.

## 1. Introduction

Renal cell carcinoma (RCC) is the most common type of urogenital cancer, being the 11th most diagnosed cancer worldwide in 2022 [1]. The annual incidence is estimated to be 430,000 new diagnoses per year, constituting 2.2% of total cancer diagnoses, with an estimated number of annual deaths of 180,000 [2]. Over the past two decades, the incidence of renal cell carcinoma (RCC) has increased by 2% per year in Europe and worldwide. The World Health Organization (WHO) classifies RCC into different subtypes based on its morphologic, molecular, and genetic features. Based on the histological features, clear cell, papillary RCC (type I and II) and chromophore are the most common solid RCC subtypes which constitute 70–90%, 10–15% and 3–5% of kidney malignancies, respectively [1]. While hereditary renal cancer is linked to somatic VHL mutations, the majority of renal cancer cases are sporadic and lack associations with specific genes. However, recent studies in cancer genomics have uncovered mutations in genes regulating epigenetics, revealing significant intra-tumor heterogeneity. These findings carry potential prognostic, predictive, and therapeutic implications [3,4].

**Figure 1:**
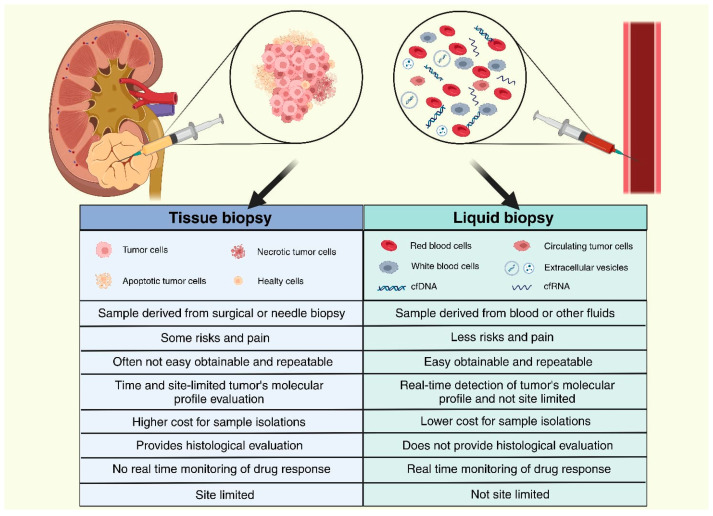
Differences between tissue and liquid biopsy.

Most patients with RCC are asymptomatic at the time of diagnosis, including patients with large tumor volumes, and diagnosis is usually made incidentally on imaging, with survival outcomes highly dependent on the stage at diagnosis [3,5]. Only 10% of patients present the classic triad of symptoms, i.e., haematuria, flank pain, and palpable masses. Other common symptoms include fever, weight loss, and leucocytosis. Approximately 20% of patients also suffer from a variety of paraneoplastic syndromes, including hypercalcemia, polycythaemia, Cushing’s syndrome, and hypertension [6]. A contrast-enhanced, triple-phase helical CT scan is the gold standard for renal masses diagnosis, distinguishing benign masses that do not require further testing from RCC. Surgery is an effective approach for managing localized tumors, while chemotherapy becomes imperative when metastasis is present at the time of diagnosis. In such cases, the 5-year survival rate is a mere 12%. For this reason, early diagnosis is essential. Nevertheless, the accuracy of the current diagnostic tools is insufficient, especially for small renal masses (SRMs) [2]. There is an urgent need for the development of a non-invasive screening tool and the identification of suitable biomarkers for RCC [7]. One potential screening method involves the use of liquid biopsy, a non-invasive examination technique that has garnered increasing attention over the years. This approach involves analysing biofluids such as blood or urine to detect the presence or absence of molecular biomarkers, offering insights into individual disease characteristics (Figure 1). This method is also used to diagnose and follow up on many other malignancies, for example, bladder cancer [8]. In RCC, potential biomarkers include circulating tumor cells (CTCs), cell-free tumor DNA (ctDNA) or circulating DNA (cfDNA), exosomes, and tumor-derived metabolites and proteins in the blood and urine.

In our research, we conducted an analysis of the current literature on liquid biopsy biomarkers for RCC, focusing on three promising biomarkers: miRNAs, exosomes, and CTCs. Our examination specifically delved into the role of liquid biopsy biomarkers in RCC from diagnostic, prognostic, and therapeutic perspectives. This approach addresses contemporary medical questions such as early diagnosis, the identification of small renal masses, and the characterization of RCC subtypes. The study also discusses the limitations of current technologies and methods in this field.

## 2. miRNAs

MiRNAs, short for microRNAs, constitute a class of small non-coding RNAs, typically composed of around 20 nucleotides. These diminutive RNA molecules play a crucial role in regulating various cellular processes, including the cell cycle, apoptosis, and differentiation. Additionally, they exert control over several metabolic pathways. Notably, numerous studies have indicated that miRNAs have the ability to modulate approximately 30–60% of human genes [9]. MiRNA genes are situated in introns or exons of coding and non-coding protein genes and have a proper transcription promoter, which could be their own or in common with other mRNAs. The encoding could be performed individually or as clusters and co-expressed with a different level of expression depending on different tissues in the same organism. The transcription of miRNA genes from the genome is facilitated by RNA polymerase II [10]. The biogenesis of miRNAs involves two distinct phases: a nuclear phase responsible for generating a primary transcript and a cytoplasmatic phase involving the processing of this primary transcript by RNase III-endonucleases DROSHA and DICER. In the cytoplasmic phase, the primary transcript is processed into small double-stranded miRNA/miRNA duplexes. These duplexes consist of two strands denoted as -3p and -5p. Subsequently, one of the two miRNA strands is incorporated into a ribonucleoprotein complex, known as the RNA-induced silencing complex (RISC) which plays a crucial function in target recognition and gene silencing. Conversely, the other strand is discarded and degraded [11]. The mechanism of the miRNA action is determined by its propensity to link the complementary sequence in the 3′-untranslated region (UTR) of mRNAs, regulating their expression by reducing mRNA stability and/or inhibiting translation. The complete or partial pairing between miRNA and target mRNA, respectively, leads to the degradation or inhibition of protein translation genes. With this mechanism of action, miRNAs are involved in cell development, viral infection, immune response, angiogenesis, and the progression of many pathologies including breast, lung, colorectal, and prostate cancer [12,13,14,15].

MiRNAs have the capability to be secreted by tumor cells into various biological fluids such as saliva, blood plasma, serum, and urine. This characteristic could be exploited for the diagnosis and prognosis prediction of oncological diseases. Once secreted, miRNAs are often bound to protein complexes or encapsulated in extracellular vesicles of different dimensions, including exosomes, microvesicles, or apoptotic bodies. This encapsulation serves to protect miRNAs from degradation operated by RNAse enzymes, providing them with a stable extracellular form. Exosomal miRNAs can influence the tumor environment at distant sites within the body, regulating gene expression and stimulating tumor growth [16].

Several studies have provided evidence of the dysregulation of circulating miRNAs into biofluids in different types of cancer. Profiling analysis of miRNAs in different human biofluids such as urine, colostrum, tears, and seminal fluid can be obtained by non-invasive techniques, highlighting a crucial aspect of the liquid biopsy method. In the context of renal cancer diagnosis and follow-up, plasma and serum are commonly used due to the stability of miRNAs within these samples. However, urine samples also offer the opportunity to monitor miRNA levels for diagnosing and monitoring the disease [17]. Cochetti et al. recently conducted a study on the diagnosis of the most common renal carcinoma, clear cell renal cell carcinoma (ccRCC), through miRNA analysis in urine [18]. The authors first investigated miRNA expression in ccRCC specimens and kidney tissues from healthy subjects through the analysis of data banks, successively validating their findings by comparing the expression of miRNAs in ccRCC and adjacent non-cancerous kidney tissue specimens by a reverse transcription–quantitative polymerase chain reaction (RT-qPCR). Successively, they developed an algorithm to identify miRNAs more likely to be present in the urine of ccRCC patients. Three miRNAs, namely miR-122, miR-1271, and miR-15b, were identified as potential markers for ccRCC diagnosis. The authors further confirmed their findings by assessing the levels of these miRNAs in the urine of 13 affected patients and 14 healthy subjects, reaching a sensitivity and a specificity of 100% (95% CI 75–100%) and 86% (95% CI 57–98%), respectively, demonstrating the potential utility of combining the expression values of specific urinary miRNAs in diagnosing ccRCC even if larger studies and more robust evidence are necessary. In another significant and similar study led by Kalogirou et al., the diagnosis of papillary renal cell carcinoma (pRCC) was performed utilizing, in this case, serum as the main source of miRNAs [19]. The authors hypothesized that the deregulation of miRNAs in malignant tissue might be reflected in serum samples, offering a non-invasive approach for the diagnosis of pRCC. Additionally, they aimed to investigate whether miRNA expression levels could be used to differentiate between type 1 and type 2 pRCC, selecting 11 differentially regulated miRNAs from the Cancer Genome Atlas (TCGA) pRCC dataset. Serum miRNA expression was determined by qRT-PCR in a total of 34 pRCC type 1, 33 pRCC type 2 and 33 control subjects across three German high-volume medical centers. The study revealed that it was not feasible to differentiate between type 1 and type 2 pRCC based on a single miRNA serum marker, encompassing both subclasses. However, the authors observed elevated levels of miR-21-5p in advanced pRCC, and diagnostic accuracy was improved by the inclusion of miR-210-3p expression. Consequently, they proposed miR-21-5p and miR-210-3p as potential biomarkers in pRCC. Another study led by G. Huang deepened the diagnostic role of serum miRNAs [20]. The work aimed to search for eligible serum biomarkers and further construct a miRNA panel with good diagnostic sensitivity or specificity. The authors enrolled 296 patients (146 RCC patients and 150 healthy controls). Serum expression levels of 30 miRNAs selected from the literature were tested by RT-qPCR in the three stages of the study (screening stage, testing stage, and validation stage). The diagnostic efficiency of miRNAs was evaluated by an ROC curve and AUC analysis. The authors finally identified a panel composed of miR-224-5p, miR-34b-3p, and miR-182-5p as the most reliable biomarker for RCC non-invasive diagnosis, due to its AUC = 0.855, demonstrating a good diagnostic efficiency.

Recent studies have highlighted the potential of miRNA dosage in fluids as a valuable tool for predicting prognosis in renal cell carcinoma (RCC). Luo et al. conducted a study aiming to identify a miRNA signature that could improve prognostic prediction for ccRCC patients [21]. Using RNA-Seq data from the TCGA database, the authors identified 177 differentially expressed miRNAs between ccRCC and paracancerous tissue. Subsequently, the ccRCC tumor samples were randomly divided into training and validation sets. A three-miRNA signature including miR130b, miR-18a, and miR-223 was constructed using the least absolute shrinkage and selection operator (LASSO) Cox regression model in the training set. Patients were then classified into high- and low-risk groups, based on the expression of the three-miRNA signature. The authors observed a significant difference in overall survival between the two groups, with a hazard ratio of 5.58 (95% CI 3.17–9.80) (*p* < 0.0001). Multivariate Cox regression analyses and subgroup analyses showed that the three-miRNA signature was an independent prognostic factor. This suggests that the three-miRNA signature could be dosed in human fluid, similar to the previously described miRNAs, to predict the overall survival of diagnosed patients.

It is important to note that while these findings show promise, further studies and stronger evidence are needed. As of now, no miRNA-based test is approved for the diagnosis and prognosis prediction of renal carcinoma. Nevertheless, the current state of the art suggests that miRNAs could play a pivotal role in the management of RCC in the near future.

## 3. Exosomes

Extracellular vesicles (EVs) can be classified into three main subtypes according to their dimensions, cellular origin, physiochemical properties, and biomolecular composition: apoptotic bodies, microvesicles, and exosomes. Exosomes, the smallest extracellular vesicles with a diameter between 30 and 150 nm, are formed through the exocytosis of multivesicular bodies, which release intraluminal vesicles upon the fusion with the plasma membrane and can carry a variety of substances, including DNA, miRNA, mRNA, cellular metabolites, and proteins [22]. The biogenesis and release of exosomes are regulated by multiple factors, including the endosomal sorting complexes required for transport (ESCRT), p53/TSAP6 pathway, Syndecan-syntenin-ALIX, Rab proteins, phospholipase D, and sphingomyelinase. Exosomal membranes are characterized by the presence of specific lipid species such as sphingomyelin, ceramide, cholesterol, and phosphatidylserine. These lipid characteristics serve as distinguishing features from other lipid-based vesicles, such as liposomes [23]. Exosomes play a crucial role as mediators of intercellular communication and are involved in facilitating organ crosstalk. Initially, their presence was overlooked, and they were colloquially termed ”cellular dust” until their potential to serve as mediators of intercellular communication and markers of various diseases, including malignancies such as kidney cancer, was recognized. Exosomes essentially represent a snapshot of the cell that produces them. When taken up by recipient cells, they can modify cell function through the cargo they carry, including various types of DNAs, RNAs, or metabolites, depending on the cell of origin and the exosome’s genesis process [24]. Exosomes can be detected in several human fluids like blood, urine, and saliva [7]. A large variety of methods are currently available for exosome isolation such as differential ultracentrifugation, size-exclusion chromatography, ultrafiltration, polyethylene glycol-based precipitation, and immunoaffinity capture, or by using microfluidics [25]. Two commonly used methods for exosome separation are differential centrifugation and the immunoaffinity method. Differential centrifugation is known for its simplicity and rapidity, but lacks the ability to distinguish other impurities like proteins or other extracellular vesicles. On the other hand, the immunoaffinity method, using antibodies or antibody-characterized magnetic beads, provides higher purity but comes with limitations related to low capture rates and high costs [26]. The unique membrane structure of exosomes offers protection against external factors such as RNases and proteases, contributing to the stability of the enclosed mRNAs, miRNAs and functional proteins. This makes exosomes highly sensitive markers for the diagnosis of RCC. A recent study led by Xuegang Wang et al. demonstrated that serum exosomal miR-210 originating from tumor tissue has the potential as a novel non-invasive biomarker for the detection and prognosis of ccRCC [27]. The study involved the examination of six miRNAs (miR-210, miR-224, miR-452, miR-155, miR-21, and miR-34a) in tissues and serum exosomes of ccRCC patients using RT-qPCR. Serum exosomal miR-210 was found to be significantly upregulated in ccRCC patients, especially at an advanced tumor stage, with a high Fuhrman grade, and in patients experiencing metastasis. Serum exosomal miR-210 may be a potential biomarker for the diagnosis (sensitivity and specificity of 82.5% and 80.0%, respectively), prognosis, and prediction of the recurrence of ccRCC.

Another interesting in vitro experimentation by Crentsil et al. corroborates exosomal miRNAs’ role as eligible biomarkers for RCC [28]. The study used the 786-O cell line (derived from ccRCC) as an in vitro ccRCC tumor model and the human renal proximal tubule cell line HK-2 as a normal renal tissue control in order to investigate the similarities of exosomal content of selected ccRCC miRNA biomarkers in the supernatant with the content of those markers in the cells themselves. The study revealed increased levels of miR-210, miR-34a, miR-155-5p, and miR-150-5p, up to two- to eight-fold, in 786-O exosomes compared with the healthy control. These miRNAs were subsequently chosen for further investigation using TaqMan RT-qPCR in addition to miR-15a and miR-205, which were selected based on their prior interest as RCC biomarkers. The study indicated that measurements of the exosomal content of miR-205 and possibly miR-150 were proportional to their respective contents in the cells that secreted them, permitting, therefore the potential dosing in vivo, serving as ccRCC biomarkers. The diagnostic potential of exosomes for RCC was also shown by C.T. Xiao et al. in a recent study that aimed to identify differences in miRNA expression profiles in peripheral blood exosomes between patients diagnosed with RCC and healthy subjects [29]. For this study, authors performed exosomal miRNA sequencing of plasma samples obtained from 5 RCC patients and 5 healthy subjects; subsequently, 22 RCC patients and 16 control subjects were investigated using qPCR to confirm the differential miRNA identified from plasma exosomal RNA sequencing. The work revealed that the expression levels of hsa-mir-92a-1-5p, hsa-mir-149-3p, and hsa-mir-424-3p were significantly abnormal in RCC patients so that they can be potentially used as biomarkers for RCC diagnosis. Exosomes have also been shown to be implicated in prognosis prediction and therapeutic response in RCC, as stated by Ivanova et al. in their 2023 study [30]. The authors focused on exosomal venous blood miRNA expression profiles of miRNAs-144, -146a, -149, -126, and -155 in 35 patients with ccRCC treated with immune checkpoint inhibitors. Expression analysis was performed using RT-qPCR and it was demonstrated that the level of microRNA-146a increased after therapy compared with the pre-therapeutic level. Conversely, the expression of miRNA-126 was reduced after the therapy with immune checkpoint inhibitors. The AUC for the miRNA-146a and miRNA-126 combination was 0.752 (95% CI 0.585–0.918), with a sensitivity of 64.3% and a specificity of 78.9%, permitting us to conclude the potential role as biomarkers, even in assessing therapy effectiveness, in RCC. In summary, exosomes, with their unique characteristics and ability to carry diverse cargo, are emerging as promising candidates for non-invasive biomarkers in the diagnosis, prognosis, and therapeutic monitoring of renal cell carcinoma. Ongoing research in this field is expected to provide additional insights and further validate the clinical utility of exosomal markers for RCC.

## 4. Circulating Tumor Cells

In patients diagnosed with metastatic cancers, the primary tumor cells have the potential to migrate to secondary sites through the bloodstream or the lymphatic system. This migration is facilitated by a biological process known as epithelial–mesenchymal transition (EMT). The initial stage of metastatic dissemination involves cancer cells invading the blood circulation. Through this process, known as “cellular seeding”, the cancer cells can disseminate to other parts of the body, establishing secondary tumor sites [31]. Indeed, during the metastatic process, circulating tumor cells (CTCs) are shed from the primary tumor into the bloodstream. Detecting and analyzing these CTCs in blood samples hold significant potential for cancer diagnosis. The presence of CTCs in the bloodstream is theoretically highly specific to the primary tumor, as these cells directly originate from the primary tumor site. Therefore, the identification and characterization of CTCs in blood samples can provide valuable information for cancer diagnosis, monitoring disease progression, and assessing treatment response [32]. However, despite these characteristics, it is not a common occurrence and mainly happens in patients with huge tumor volumes. The absence of CTCs in small tumors and, therefore, in the initial stages of the disease, limits their diagnostic applicability. Additionally, CTC detection in the blood is a complex process due to the similarities of CTCs to white blood cells, which have similar cellular shapes and from which they have to be distinguished. Nevertheless, it has been observed that both these cell populations are significant to monitor the prognosis of RCC. As reported in a recent study by Yibing Guan et al., total CTCs and circulating tumor-cell-associated white blood cell (CTC-WBC) clusters, along with measures of cancer dimensions, were collectively evaluated as prognostic factors for the clinical outcomes of RCC patients. The study, which included a total of 163 RCC cases, revealed a higher count of CTCs and CTC-WBC clusters, coupled with a larger solid tumor diameter, served as negative prognostic factors associated with a detrimental impact on metastasis-free survival. The research demonstrated a negative correlation between the number of CTCs, solid cancer diameter, and the overall survival of RCC patients [33]. Different strategies are used to enrich the CTC population, with methods based on their mechanical features (i.e., cellular size and density) as well as methods relying on antibodies or fluorescence in situ hybridization (FISH). Mechanical features, such as cell size [34], are used in several tests. However, it has been observed that these characteristics often overlap with those of white blood cells [35]. To address this issue, more precise techniques have been developed and one of the most commonly used is antibody-based enrichment which relies on the presence or absence of specific antigens on the cell surface, facilitating positive or negative selection of cell populations. An example of a negative selection strategy is the use of the specific leukocyte marker CD45 and its corresponding antibody to deplete white blood cells from the sample and consequently enrich the CTC population [36]. Conversely, a method for positive enrichment of CTCs involves the use of immunomagnetic beads or nanochips coated with antibodies targeting common CTC markers. Among these markers, the epithelial cell adhesion molecule (EPCAM) stands out and is the only marker approved by the FDA for the diagnosis of breast, colon, and prostate cancer [37,38]. After CTCs have been distinguished and isolated, they can be used to diagnose and follow up several cancers such as colorectal, bladder, lung, and prostate cancer [39,40,41,42]. Despite the scarcity of data regarding the use of CTCs for renal cancer diagnosis, a recent study suggested that the baseline number of isolated CTCs could serve as a predictive factor for prognosis in patients diagnosed with metastatic RCC [43]. The authors concluded that the presence of three or more CTCs per millilitre at the baseline is associated with significantly shorter progression-free survival and overall survival in patients with metastatic RCC and treated with an antiangiogenic tyrosine kinase inhibitor as a first-line regimen, in particular, sunitinib, pazopanib, and sorafenib. However, it is crucial to acknowledge that other factors, such as the age of the patients and the chosen first-line therapy, can also influence survival outcomes in malignancies [44]. The prognostic role of CTCs has been also deepened by Z.L. Wang et al. in a study investigating the relationship of dynamic changes of CTCs and Beclin-1 expression of CTCs with renal cell carcinoma (RCC) prognosis [45]. Beclin-1 is an autophagy gene that could be more or less expressed by CTCs. The authors enrolled a total of 69 patients with RCC and divided them into two groups based on the postoperative status of distant metastasis, including the metastasis-free group (n = 58) and the metastatic group (n = 11). The patients received multiple CTC tests and peripheral blood samples were obtained at three different time points (1 day before operation, 6 months and 12 months after operation) for this study. CTCs were divided into epithelial, mesenchymal and mixed phenotypes based on different surface biomarkers. There were no significant differences in initial CTC counts between the metastasis-free group and the metastatic group. For the metastatic group, the number of mixed CTCs at the third time point (12 months) was significantly higher than that of mixed CTCs preoperatively and at the second time point (6 months). In the metastatic group, the number of Beclin1 positive CTCs was significantly higher than that of Beclin1 negative CTCs preoperatively (*p* < 0.05); moreover, there were several significant changes in Beclin1 positive CTCs with different types and at different time points. The authors thus concluded that the recurrence or metastasis of RCC was related to the variation trend of CTCs, especially mesenchymal CTCs and Beclin1-positive CTCs.

Currently, there are no approved diagnostic assays specifically designed for renal cancer, primarily due to the absence of well-established and specific markers for RCC and its various subtypes. Consequently, CTCs could potentially play a significant role in the future, particularly in the realms of follow-up care and therapeutic selection. Additionally, CTCs hold promise for predicting outcomes and prognosis in individuals with renal cancer (Table 1).

## 5. Circulating Tumor DNA

Circulating tumor DNA (ctDNA) has emerged as a promising avenue for both diagnosis and monitoring of RCC, imposing as a novel and important element of liquid biopsy, permitting it to offer a non-invasive approach complementing imaging techniques. ctDNA, which consists of fragmented DNA shed by tumor cells into the bloodstream, reflects the genetic landscape of the tumor and could provide valuable insights into its molecular profile, permitting us, theoretically to also detect specific genetic alterations such as mutations in the von Hippel-Lindau (VHL) gene or alterations in genes associated with the mTOR pathway [46,47]. The efficiency and the consistency of ctDNA are impacted by the technique chosen to isolate ctDNA itself, which could be grossly divided into non-commercial methods, utilizing time-consuming and highly efficient extraction methods and commercial methods which instead rely on higher repeatability and reproducibility at the cost of a lower yield in terms of ctDNA extracted [48,49]. In the early analysis era of ctDNA in RCC, five studies investigated the presence of ctDNA in RCC patients versus healthy controls, utlizing tumor tissue DNA as a guide, delivering interesting data. Bettegowda et al., in a study involving different cancer types, identified in five patients with RCC, ctDNA in only two cases [50]. Similarly, Corrò et al. identified ctDNA only in one patient in a total of nine [51]. Despite the limited detection rate, the data were further confirmed in successive studies on metastatic and non-metastatic patients, also aided by the introduction of deep sequencing analysis, delivering a detection rate of 42–54% [52,53,54]. The possibility to utilize targeted sequencing of plasma, i.e., the application of targeted panel sequencing of DNA on plasma, avoiding prior analysis of tumor tissue DNA, permitted the identification of newly acquired mutations, at the cost of being less sensitive compared to tumor-guided analysis. Most of these studies were performed on patients with metastatic disease and reported a ctDNA detection rate of over 50%; therefore, most of the authors recognized a correlation between tumor burden and ctDNA detection [55,56,57]. The correlation was confirmed by Bacon et al., who found a relatively low detection rate of 33% in patients in which the plasma samples were collected after the surgical removal of the primary tumor [58]. Beyond diagnosis, ctDNA holds considerable promise for monitoring disease progression and treatment response in RCC patients. By tracking changes in the abundance and genetic composition of ctDNA over time, tumor dynamics could be analyzed and therefore identify opportunities for therapeutic intervention. Additionally, ctDNA analysis allows for the early detection of disease recurrence or metastasis, enabling timely adjustments to treatment plans and potentially improving patient outcomes. In this regard, Smith et al. observed that the level of ctDNA increased before the initiation of the treatment and decreased with response to the treatment to further increase with disease progression or lack of response to the treatment [52]. Similar effects were observed by Lasseter et al., and Yamamoto et al., who investigated ctDNA in their patients longitudinally [54,59]. Interestingly, the same studies also reported that ctDNA detection in patients with various stages of RCC was significantly associated with either risk of death or shorter progression-free, cancer-specific and overall survival [59,60,61]. In a recent study by Kim et al., ctDNA was utilized to evaluate the response of patients with metastatic RCC treated with immune checkpoint inhibitors, reporting the decrease in ctDNA in those who responded to the treatment as well as in those showing partial response while ctDNA increased in those showing disease progression [62]. More recently, the role of ctDNA in RCC has evolved from the diagnosis and monitoring of treatment to a predicting role. In particular, in a study by Park et al. involving 48 patients who underwent partial nephrectomy for T1a disease, the authors aimed to predict the upstaging to T3a disease. Indeed, ctDNA was detected in over 75% of patients who reported an upstaging compared to 2.8% of patients with confirmed T1a disease [63]. The utilization of ctDNA as a non-invasive biomarker for RCC is still progressing and, with its rapid, cost-effective and minimally invasive nature, is bound to be more and more implemented in different stages of RCC disease, from early diagnosis to progression prediction and the monitoring of treatment. Different ongoing studies aim to analyze the possibilities related to ctDNA in RCC in an effort to propel the translation of research into routine clinical practice [64]. Challenges remain in terms of optimizing technical assays, ensuring high-quality output, and refining library preparation procedures in order to achieve sufficient sequencing depth for precise analyses, especially in methylation profiling.

## 6. Conclusions

Despite advancements, RCC diagnosis remains challenging, especially for small renal masses. The urgency for non-invasive screening tools and biomarkers led to the exploration of liquid biopsy, focusing on miRNAs, exosomes, and CTCs. MiRNAs, small non-coding RNAs, exhibit dysregulation in RCC, offering diagnostic and prognostic potential. Several studies highlight their utility in different biofluids, including plasma, serum, and urine, for RCC detection and subtype differentiation. Promising miRNA signatures have been identified, correlating with overall survival, emphasizing their future role in RCC management. Similarly, exosomes have emerged as promising biomarkers due to their ability to carry diverse cargo, including miRNAs, while CTCs, shedding from primary tumors into the bloodstream, offer insights into cancer biology. While these liquid biopsy approaches hold promise, their clinical translation necessitates further validation and standardization. Challenges include the need for larger studies, robust evidence, and the identification of specific markers. Currently, there are no approved diagnostic assays for renal cancer, highlighting the potential future applications of liquid biopsy in follow-up care, therapeutic selection, and outcome prediction in RCC patients. Ongoing research is expected to refine these biomarkers and enhance their clinical utility in the comprehensive management of renal cell carcinoma.

## Figures and Tables

**Table 1:** Summary of biomarkers and studies in RCC.

Biomarker	Region	Year	Detected Abnormality	Cohorts	Practice in Clinical	Results	Ref.
Urinary miRNAs	Italy	2020	miR-122, miR-1271, and miR-15b overexpression	13 ccRCC-diagnosed patients and 14 healthy controls	Diagnosis	miR-122, miR-1271, and miR-15b candidate as potential biomarkers for ccRCC diagnosis	[18]
Serum miRNAs	Germany	2020	miR-21-5p and miR-210-3p overexpression	34 pRCC type 1, 33 pRCC type 2 and 33 control subjects	Diagnosis	miR-21-5p and miR-210-3p candidates as potential biomarkers for pRCC diagnosis. Impossibility to make a differential diagnosis between pRCC type 1 and 2 based on miRNA expression	[19]
Serum miRNAs	China	2020	miR-224-5p, miR-34b-3p and miR-182-5p overexpression	146 RCC patients and 150 healthy controls	Diagnosis	A panel formed by miR-224-5p, miR-34b-3p and miR-182-5p candidate as the most reliable biomarker for RCC non-invasive diagnosis	[20]
miRNAs expressed by ccRCC and paracancerous tissue	China	2019	miR130b, miR-18a, and miR-223 overexpression	544 ccRCC tumor specimens and 71 adjacent nontumor renal specimens	Prognosis prediction	Multivariate Cox regression analysis and subgroup analysis showed that the three-miRNA signature was an independent prognostic factor that could be used to predict the prognosis of ccRCC patients by dosing the three miRNAs in biofluid	[21]
Exosomal miRNAs	China	2018	miR-210, miR-224, miR-452, miR-155, miR-21, and miR-34a expression	45 patients diagnosed with ccRCC	Diagnosis, prognosis and recurrence prediction	Serum exosomal miR-210 may be a potential biomarker for the diagnosis, prognosis, and prediction of the recurrence of ccRCC, especially for metastatic ccRCC	[27]
Exosomal miRNAs	USA	2018	miR-210, MiR-34a, miR-155-5p and miR-150-5p overexpression	In vitro (786-O cell line (derived from ccRCC) as an in vitro ccRCC tumor model and the human renal proximal tubule cell line HK-2 as a normal renal tissue control)	Diagnosis	Exosomal content of miR-205 and possibly miR-150 were proportional to their respective contents in the cells that secreted them, serving as ccRCC biomarkers	[28]
Exosomal miRNAs	China	2020	hsa-mir-92a-1-5p, hsa-mir-149-3p and hsa-mir-424-3p abnormal expression	22 RCC patients and 16 controls	Diagnosis	hsa-mir-92a-1-5p, hsa-mir-149-3p and hsa-mir-424-3p can be potentially used as plasmatic biomarkers for RCC diagnosis	[29]
Exosomal miRNAs	Russia	2023	miRNAs-144, -146a, -149, -126, and -155 expression	35 RCC patients treated with immune checkpoint inhibitors	Prognosis prediction	miRNA-146a and miRNA-126 combined expression showed a potential role as biomarkers, even in assessing therapy effectiveness, in RCC	[30]
Exosomal miRNAs	Italy	2021	correlation between CTC counts and progression-free survival (PFS) in patients with metastatic RCC treated with an antiangiogenic tyrosine kinase inhibitor as a first-line regimen	195 patients treated at the baseline with pazopanib or sunitinib	Prognosis prediction	the baseline number of isolated CTCs could serve as a predictive factor for prognosis in patients diagnosed with metastatic RCC	[43]
Exosomal miRNAs	China	2018	the relationship of dynamic changes of CTCs and Beclin-1 expression of CTCs with renal cell carcinoma (RCC) prognosis	69 patients diagnosed with RCC and treated with surgery [metastasis-free group (n = 58) and metastatic group (n = 11)]	Prognosis prediction	recurrence or metastasis of RCC was related to the variation trend of CTCs, especially mesenchymal CTCs and Beclin1-positive CTCs	[45]

## Data Availability

Data derived from public domain resources.
